# A meta‐analysis assessing reliability of the Yale Food Addiction Scale: Implications for compulsive eating and obesity

**DOI:** 10.1111/obr.13881

**Published:** 2024-12-23

**Authors:** Haitham Jahrami, Waqar Husain, Khaled Trabelsi, Achraf Ammar, Seithikurippu R. Pandi‐Perumal, Zahra Saif, Marc N. Potenza, Chung‐Ying Lin, Amir H. Pakpour

**Affiliations:** ^1^ Government Hospitals Manama Bahrain; ^2^ Department of Psychiatry, College of Medicine and Medical Sciences Arabian Gulf University Manama Bahrain; ^3^ Department of Humanities COMSATS University Islamabad Islamabad Pakistan; ^4^ High Institute of Sport and Physical Education of Sfax University of Sfax Sfax Tunisia; ^5^ Research Laboratory: Education, Motricity, Sport and Health, EM2S, LR19JS01 University of Sfax Sfax Tunisia; ^6^ Department of Training and Movement Science Institute of Sport Science Mainz Germany; ^7^ Research Laboratory, Molecular Bases of Human Pathology, LR19ES13, Faculty of Medicine of Sfax University of Sfax Sfax Tunisia; ^8^ Centre for Research and Development Chandigarh University Mohali Punjab India; ^9^ Division of Research and Development Lovely Professional University Phagwara Punjab India; ^10^ Department of Psychiatry and Neuroscience and the Child Study Center and Wu Tsai Institute Yale School of Medicine/Yale University New Haven CT USA; ^11^ Institute of Allied Health Sciences, College of Medicine National Cheng Kung University Tainan Taiwan; ^12^ Department of Nursing, School of Health and Welfare Jönköping University Hälsohögskolan Jönköping Sweden

**Keywords:** Cronbach's alpha, food addiction, meta‐analysis, reliability

## Abstract

Food addiction (FA) is linked to eating disorders and obesity. The Yale Food Addiction Scale (YFAS), which has various versions in different languages, is widely used to assess FA worldwide. This meta‐analysis aimed to assess the YFAS through reliability generalization meta‐analysis (REGEMA). From their inception until April 2024, a comprehensive systematic review across more than 30 databases was conducted to identify studies reporting reliability measures (e.g., Cronbach's alpha and McDonald's omega) of the YFAS. Sixty‐five studies were included in this meta‐analysis, with a median sample size of 451 participants. The results of the random‐effects meta‐analysis showed a high pooled reliability coefficient (*α* = 0.85, 95% CI: 0.83 to 0.86 *p* < 0.001). Test–retest reliability was also estimated using a random‐effects meta‐analysis of 10 studies, resulting in a pooled test–retest correlation coefficient of intraclass coefficients of (*ICC* = 0.77, 95% CI: 0.70 to 0.84, *p* < 0.001). These findings highlight the consistency and robustness of the YFAS in detecting FA across studies, suggesting its reliability for screening for FA‐related disordered eating.

## 1 INTRODUCTION

Obesity and overeating are important public health issues worldwide,^1^ with increases in global obesity rates generating serious health problems.^2^, ^3^ The increased availability and inexpensive nature of manufactured calorie‐dense foods may underlie increased caloric consumption that may constitute food addiction (FA). FA implies that some people may react to certain highly processed foods, such as chips, chocolate, pizza, and burgers, similarly to drugs.^4^, ^5^ Hyperpalatable foods, especially those heavy in carbohydrates, sugar, fat, or salt, may be addictive, generating cravings and difficulties in managing food consumption.^4^ FA has been assessed using diagnostic criteria similar to those used for drug addiction.^6^, ^7^, ^8^ Similarities between food intake and drug use suggest that particular meals or food additives may stimulate addictive processes, making it difficult to eat in healthier fashions.^9^, ^10^ Intermittent sugar consumption in preclinical models may cause addiction‐like responses such as tolerance, withdrawal, and repeated use.^11^ FA and drug abuse may involve comparable brain pathways, including dopamine and opiate systems.^12^ Food and addictive drugs may promote dopamine release linked to perceived reward values.^13^ Opioid‐receptor antagonists may reduce alcohol and high‐fat sweet cravings.^14^, ^15^ From animal models to human neuroimaging studies, FA and substance use disorders established in psychiatric nomenclature systems share biological and behavioral features.^16^, ^17^, ^18^ Neuroimaging studies have identified overlapping brain areas responsive to food and drugs, with similar neural underpinnings.^19^, ^20^

Psychological aspects of FA have also been studied extensively.^21^, ^22^, ^23^, ^24^ FA has been linked to genetic predispositions for addictive disorders, dysfunctional reward processing, emotional eating as a coping mechanism, and craving in humans.^18^, ^25^, ^26^, ^27^ Bulimia, an eating disorder, also involves overeating.^28^, ^29^ Personality features such as alexithymia, which involves limited emotional awareness, have been connected to addictions.^30^, ^31^ Individuals with obesity and FA may have more alexithymia and difficulty regulating emotions, which may worsen addictive eating behaviors.^32^

Measuring food addiction is crucial for understanding its role in obesity and eating disorders. Studies have highlighted the association between food addiction, binge eating disorder (BED), and obesity.^33^, ^34^, ^35^ Tools like the Yale Food Addiction Scale (YFAS) and the modified Yale Food Addiction Scale 2.0 (mYFAS2.0) have been developed to assess food addiction prevalence, with findings indicating a significant prevalence in the general population, especially among females and younger individuals.^36^, ^37^ The discriminant validity of these measures is pivotal to differentiate between food addiction and other eating disorders such as BED. This provides valuable insights into the distinct clinical profiles associated with food addiction symptoms.^33^, ^35^ Understanding the clinical profile of food addiction and its relationship with obesity and eating disorders can aid in early diagnosis, intervention, and management.^33^, ^35^

The Yale Food Addiction Scale (YFAS) and its variants are the most used measures of FA.^28^ This scale adapts criteria from the Diagnostic and Statistical Manual of Mental Disorders.^38^ for substance use disorders (initially substance dependence from the fourth edition, DSM‐IV) to problematic consumption of palatable foods, including impaired control, tolerance, and withdrawal symptoms. At least three of seven symptoms of FA reflect clinically relevant impairment or suffering.^28^, ^39^ The YFAS has also been appreciated for linking several addiction factors, such as increased consumption in response to emotional and environmental cues in adults.^27^, ^28^, ^40^, ^41^, ^42^ The YFAS is also associated with obesity and other deleterious effects of overeating.^28^, ^43^, ^44^, ^45^ Thus, the YFAS appears to be a valid FA screening instrument. It has been validated in multiple languages, permitting cross‐cultural assessments.^43^, ^46^, ^47^, ^48^ The modified YFAS (mYFAS), a nine‐question self‐reported assessment, is also reliable and valid, similar to the full YFAS.^49^, ^50^, ^51^ The YFAS 2.0 was updated to match DSM‐5 diagnostic criteria for substance use disorders, including severity specifiers and desire.^51^ The YFAS 2.0 has demonstrated better internal consistency than the original YFAS and is replacing its original version in FA investigations.^51^ The modified YFAS 2.0 (mYFAS 2.0) containing 11 items was developed for use as a brief screening instrument in large epidemiological samples.^52^ The YFAS‐C^53^ and YFAS‐C 2.0^54^ were developed specifically for children.

Apart from its validity, which has been established in various studies, the reliability of the YFAS (including its variations in the mYFAS, YAFS‐C, YFAS 2.0, and mYFAS 2.0) may vary across studies due to sample characteristics, cultural differences, and administration or scoring methods. The objective of the current study was to analyze a variety of studies using YFAS to determine the reliability of the YFAS across demographics and regions. The reliability of an instrument is of extreme significance to trust results across studies.^55^ Reliability generalization meta‐analysis (REGEMA) is a useful technique for evaluating instrument efficacy and consistency. This technique facilitates researchers to evaluate scale/measure reliability across contexts, people, and settings by synthesizing data from several investigations using a specific instrument. It also identifies patterns of consistency or variability in the performance of the instrument across studies to evaluate its reliability. REGEMA further allows the researchers to assess how sample characteristics, administration techniques, and cultural variations may affect instrument reliability. REGEMA also helps identify the sources of measurement errors and reliability variables of the instrument. By carefully assessing multiple studies using the same instrument, REGEMA helps researchers estimate reliability and find ways to improve the instrument's psychometric features.^56^ In the current study, we hypothesized that YFAS and its variants would have acceptable reliability if tested across cultures and languages. Therefore, in the current meta‐analysis, we intended to estimate the reliability of YFAS and its variants through REGEMA.

## 2 METHODS

### 2.1 Literature search and inclusion criteria

The electronic literature search for this meta‐analysis included over 30 databases and sources, with searches across 19 major indexing and abstracting services: BibCnrs, CNKI, CNPIEC, Digital Science, DOAJ, EBSCO, Scopus (Elsevier Databases), Gale, PubMed/MEDLINE, PMC (National Library of Medicine), OpenAIRE, ProQuest, PSYNDEX, SafetyLit, SSCI (Web of Science), Current Contents ‐ Social and Behavioral Sciences (Web of Science); five journal ranking lists: ANVUR, Journal Citation Reports, Norwegian Register for Scientific Journals, Publication Forum ‐ JUFO (Federation of Finnish Learned Societies), SCImago Journal and Country Rank; four journal directories: Electronic Journals Library (EZB), JournalGuide, Hinari (Research4Life), OARE (Research4Life); two repository delivery services: DeepGreen, Jisc; two digital preservation services: CLOCKSS, Swiss National Library (Helveticat); and three content aggregators: Google Scholar, Scilit, WorldCat (OCLC).

We used keyword terms such as “Yale Food Addiction Scale,” “YFAS,” and “reliability.” We sought studies in any discipline published in any language that reported a reliability estimate (e.g., Cronbach's alpha, McDonald's omega … etc.) for at least one version of the YFAS. The search covered literature from the measure's inception through April 2024.

### 2.2 Coding procedures and variables

Two authors (HJ and WH) coded sample and study characteristics from each report, including YFAS version, sample size, population type, age, sex composition, clinical status, and reliability type. We calculated interrater agreement statistics and resolved discrepancies through consensus discussion.

### 2.3 Meta‐analytic approach

Reliability coefficients were used as the effect‐size metric. A random‐effects model was employed to estimate the average correlation and account for potential heterogeneity in effects across studies. Heterogeneity was assessed using Cochran's Q test and the *I* ^*2*^ statistic. Prediction intervals, which estimate the distribution of true effects around the average, were calculated to evaluate the impact of heterogeneity. The DerSimonian‐Laird estimator was used to estimate the heterogeneity variance using *τ and τ* ^*2*^. Analyses were conducted using Knapp‐Hartung adjustments to calibrate confidence intervals and significance tests. Potential outliers and influential cases were identified by examining studentized residuals and Cook's distance values, respectively.

In this review, we reported raw Cronbach's alpha values and their equivalents (such as the McDonald's omega Kuder and Richardson Formula 20 test) without applying Fisher's r‐to‐z transformation. Although meta‐analysts use the Fisher transformation sometimes to stabilize variance when pooling correlation coefficients across studies, particularly when studies exhibit significant variance, we determined this approach was unnecessary in our case.^57^ We chose raw reliability coefficient values for the ease of comprehension and direct interpretability for readers familiar with reliability coefficients. This decision also facilitates straightforward comparisons with other studies in the field that typically report untransformed alpha values. Additionally, the consistency in methodologies and sample characteristics across the included studies in our meta‐analysis reduced the need for variance stabilization.^57^ Finally, we conducted a sensitivity analysis comparing the results of meta‐analyses using raw and Fisher‐transformed coefficient values, revealing no substantial differences in the overall conclusions, with discrepancies of no more than 2%.

We conducted meta‐regression analyses that considered age and sex, as well as subgroup analyses based on population type and language, to explore potential moderators of reliability. Funnel plots and regression‐based tests (Egger's regression, rank correlation) were used to evaluate publication bias. In cases where bias was indicated, publication bias adjustments were applied, including the trim‐and‐fill imputation method to estimate an unbiased effect, as well as p‐uniform publication bias tests. The fail‐safe N was calculated to determine the number of missed null studies that would nullify the results. Excess significance testing evaluated whether the observed significant findings exceeded expectations. Effect size robustness was examined through p‐curve analysis, estimating effect sizes and adjusting for potential p‐hacking. All analyses used a two‐tailed *α* = 0.05 significance threshold unless otherwise specified.

## 3 RESULTS

**FIGURE 1:**
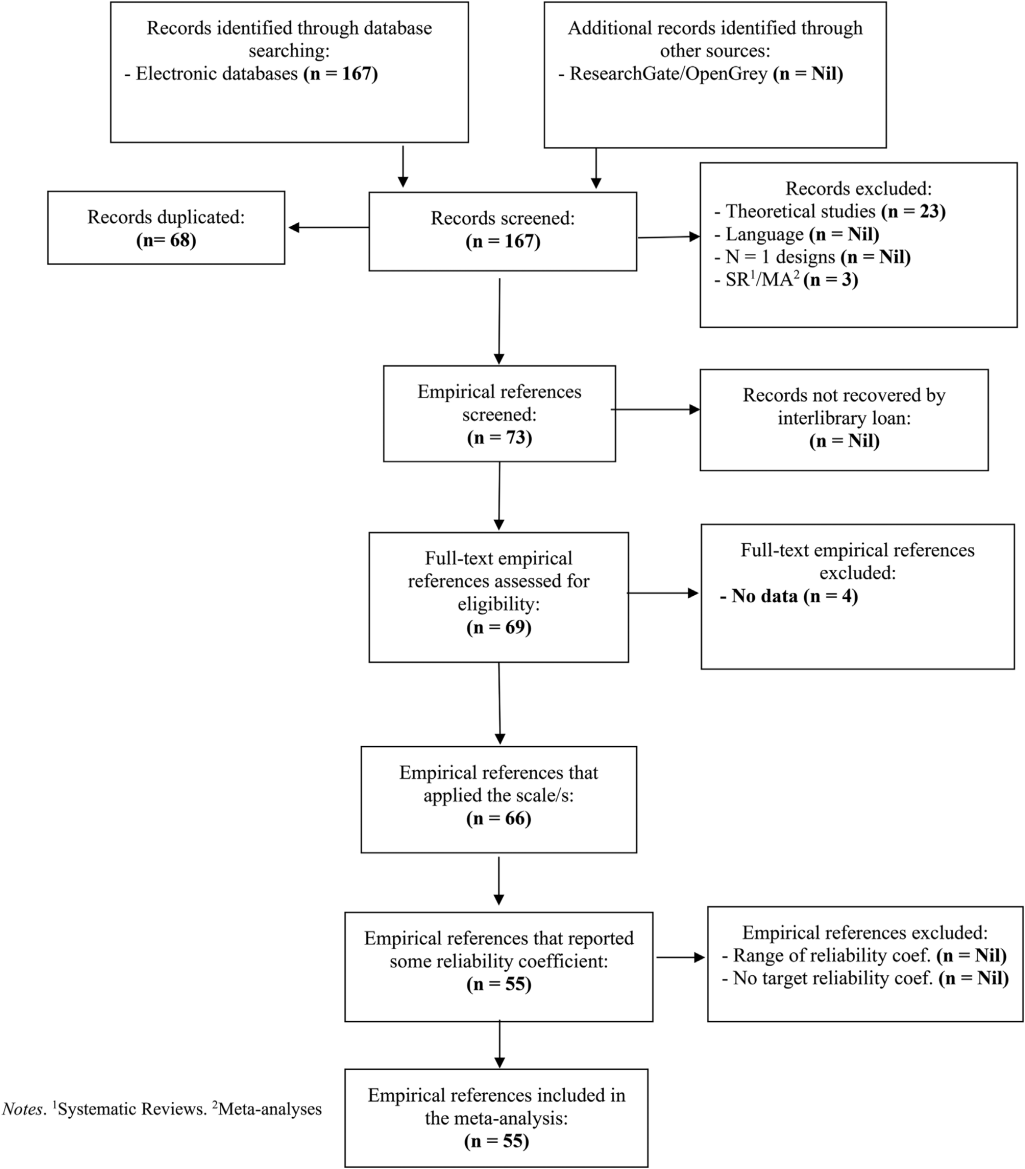
REGEMA flow diagram for study selection.

The meta‐analysis selection process involved a comprehensive search of electronic databases, resulting in the identification of 167 records. These records were then screened for eligibility, and after removing duplicates (*n* = 68), theoretical studies (*n* = 23), and records related to SR1/MA2 (*n* = 3), a total of 73 empirical references remained. Additional sources such as ResearchGate and OpenGrey did not yield any relevant records. Interlibrary loan did not recover any additional records. Of the 73 screened empirical references, 69 full‐text references were assessed for eligibility. Among them, four references were excluded as they did not provide any data. The remaining 65 references, which applied the scale/s under investigation, were included in the meta‐analysis. Among the included references, 55 reported some reliability coefficient, and the 55 published reports contained 65 studies (Figure 1, Table 1).

**TABLE 1:** Descriptive information of the included studies regarding the Yale Food Addiction Scale (YFAS) and its variants.

SN	Study	Ref.	Version	Language	Sample n	Sample type	Age	%male	Metric IC	IC	Metric TRT	TRT
1	Santos Flores J.M., 2024	[61]	YFAS‐C	Spanish	448	NC	NR	NR	Alpha	0.78	NR	NR
2	Niroumand Sarvandani M., 2024	[58]	mYFAS 2.0	Persian	9606	NC	29.61	19.3	Alpha	0.89	NR	NR
3	Horsager C., 2023	[68]	YFAS‐C 2.0	Danish	972	M	15.15	40.65	Kuder–Richardson 20	0.81	NR	NR
4	Li R., 2023	[78]	YFAS 2.0	Finnish	360	NC	32.5	34.44	Kuder–Richardson 20	0.72	NR	NR
5	Gonçalves S., 2022	[69]	YFAS 2.0	Portuguese	302	NC	21.37	0	Alpha	0.95	NR	NR
6	Li S., 2022	[79]	mYFAS 2.0	Simplified Chinese	1132	NC	19.33	38.4	Kuder–Richardson 20	0.82	*ICC*	0.75
7	Hallit S., 2022	[82]	mYFAS 2.0	Arabic	1268	NC	26.18	34.9	Alpha	0.85	NR	NR
8	Saffari M., 2022 (mYFAS 2.0)	[73]	YFAS 2.0	Traditional Chinese	974	NC	23.7	40.7	Alpha	0.91	NR	NR
9	Saffari M., 2022 (YFAS 2.0)	[73]	mYFAS 2.0	Traditional Chinese	974	NC	23.7	40.7	Alpha	0.82	NR	NR
10	Ghanbari N., 2022	[73]	YFAS 2.0	Persian	451	NC	25.8	35	Alpha	0.93	NR	NR
11	Chen I.‐H., 2022 (YFAS 2.0)	[81]	YFAS 2.0	Traditional Chinese	687	NC	24	40.8	Alpha	0.9	*ICC*	0.71
12	Chen I.‐H., 2022 (mYFAS 2.0)	[81]	mYFAS 2.0	Traditional Chinese	687	NC	24	40.8	Alpha	0.89	*ICC*	0.69
13	Pipová H., 2021	[84]	mYFAS 2.0	Czech	3950	NC	15.73	46	Kuder–Richardson 20	0.86	NR	NR
14	Horsager C., 2021	[76]	YFAS‐C 2.0	Danish	576	NC	14.8	44.4	Alpha	0.8	NR	NR
15	Benítez Brito N., 2021	[62]	YFAS‐C	Spanish	197	NC	NR	46	Alpha	0.8	NR	NR
16	Zhang H., 2021	[80]	mYFAS 2.0	Simplified Chinese	1099	NC	20	21	Kuder–Richardson 20	0.84	NR	NR
17	Moghaddam S.A.P., 2021	[70]	YFAS	Persian	450	C	39.52	0	Kuder–Richardson 20	0.86	NR	NR
18	Manzoni G.M., 2021 (Clinical Sample)	[85]	YFAS 2.0	Italian	400	C	55.54	44	Kuder–Richardson 20	0.874	NR	NR
19	Manzoni G.M., 2021 (General Population)	[85]	YFAS 2.0	Italian	304	NC	34.26	29.9	Kuder–Richardson 20	0.874	*ICC*	0.853
20	Lin C.‐Y., 2021	[74]	YFAS‐C	Persian	1186	C	15.5	56.15	Kuder–Richardson 20	0.81	*ICC*	0.83
21	Haghighinejad H., 2021	[75]	YFAS 2.0	Persian	330	NC	33.3	34.8	Alpha	0.81	NR	NR
22	Pipová H., 2020	[63]	YFAS 2.0	Czech	1841	NC	NR	48.8	Alpha	0.89	NR	NR
23	Brunault P., 2020 (Sample 1)	[88]	mYFAS 2.0	French	250	NC	28.4	20	Kuder–Richardson 20	0.78	NR	NR
24	Brunault P., 2020 (Sample 2)	[88]	mYFAS 2.0	French	345	C	43.4	24.3	Kuder–Richardson 20	0.73	NR	NR
25	Panahi A., 2020	[64]	YFAS 2.0	Persian	180	M	NR	24.8	Alpha	0.87	NR	NR
26	Druk I.V., 2020	[60]	YFAS 2.0	Russian	25	M	29.5	40	Alpha	0.82	NR	NR
27	Horsager C., 2020	[77]	YFAS 2.0	Danish	1699	NC	43.5	41.3	Kuder–Richardson 20	0.86	NR	NR
28	Cardoso T.Q., 2020	[65]	mYFAS 2.0	Portuguese	150	NC	NR	NR	Alpha	0.91	NR	NR
29	Nantha Y.S., 2020	[90]	YFAS	Malay	358	C	32.37	32.7	Kuder–Richardson 20	0.8	NR	NR
30	Linardon J., 2019	[97]	YFAS 2.0	English	220	M	29.54	6	Kuder–Richardson 20	0.86	NR	NR
31	Kim J.H., 2019	[53]	YFAS‐C	Korean	419	NC	13.74	44.1	Kuder–Richardson 20	0.69	NR	NR
32	Khine M.T., 2019	[92]	YFAS 2.0	Japanese	731	NC	20.8	21.3	Kuder–Richardson 20	0.78	NR	NR
33	Buyuktuncer Z., 2019	[93]	YFAS	Turkish	1033	NC	31.1	42.49	Alpha	0.83	NR	NR
34	Imperatori C., 2019	[86]	mYFAS 2.0	Italian	262	NC	29.43	29.78	NR	0.91	NR	NR
35	Nunes‐Neto P.R., 2018	[59]	mYFAS 2.0	Portuguese	7639	NC	27.2	28.7	Alpha	0.89	NR	NR
36	Schiestl E.T., 2018	[98]	YFAS‐C 2.0	English	127	NC	14.28	48.03	Alpha	0.82	NR	NR
37	Fawzi M., 2018	[83]	YFAS 2.0	Arabic	236	NC	19.1	46.6	Kuder–Richardson 20	0.89	*ICC*	0.95
38	Granero R., 2018	[71]	YFAS 2.0	Spanish	453	M	31	22.07	Alpha	0.8	NR	NR
39	Manzoni G.M., 2018	[42]	YFAS	Italian	256	NC	23.93	21.9	Kuder–Richardson 20	0.72	NR	NR
40	Erzsébet Magyar É., 2018	[95]	YFAS‐C	Hungarian	191	NC	15.1	57	Kuder–Richardson 20	0.82	NR	NR
41	Meule A., 2017 (Clinical Sample 1)	[96]	YFAS 2.0	German	44	C	27.27	11	Alpha	0.9	NR	NR
42	Meule A., 2017 (General Population Sample 1)	[96]	YFAS 2.0	German	411	NC	25.38	11	Alpha	0.9	NR	NR
43	Meule A., 2017 (Clinical Sample 2)	[96]	YFAS 2.0	German	63	C	39.83	21.7	Alpha	0.87	NR	NR
44	Meule A., 2017 (General Population Sample 2)	[96]	YFAS 2.0	German	70	NC	39.61	21.7	Alpha	0.87	NR	NR
45	Aloi M., 2017	[87]	YFAS 2.0	Italian	574	NC	21.42	43	Alpha	0.87	NR	NR
46	Brunault P., 2017	[89]	YFAS 2.0	French	330	NC	28.9	19.7	Kuder–Richardson 20	0.83	NR	NR
47	Torres S., 2017	[45]	YFAS	Portuguese	190	C	43.21	12.6	Alpha	0.93	NR	NR
48	Torres S., 2017	[45]	YFAS	Portuguese	278	NC	23.06	33.5	Alpha	0.88	NR	NR
49	Carr M.M., 2017	[66]	mYFAS 2.0	English	923	M	NR	14.3	Alpha	0.86	NR	NR
50	Schulte E.M., 2017	[99]	mYFAS 2.0	English	213	NC	33.68	28.6	Kuder–Richardson 20	0.86	NR	NR
51	Lemeshow A.R., 2016	[50]	YFAS	English	232	NC	NR	41.6	Alpha	0.84	Kappa	0.73
52	Lemeshow A.R., 2016	[50]	mYFAS 2.0	English	232	NC	NR	41.6	Alpha	0.67	Kappa	0.79
53	Gearhardt A.N., 2016	[51]	YFAS 2.0	English	550	NC	33.84	45.7	Kuder–Richardson 20	0.9	NR	NR
54	Swarna Nantha Y., 2016	[91]	YFAS 2.0	Malay	250	C	38.9	43.2	Kuder–Richardson 20	0.76	NR	NR
55	Moreno M.I.V., 2016	[72]	YFAS	Spanish	37	NC	14	23	Alpha	0.79	*ICC*	0.56
56	Pursey K.M., 2016	[100]	YFAS	English	69	NC	25.3	5.8	Alpha	0.86	*ICC*	0.71
57	Chen G., 2015	[47]	mYFAS 2.0	Simplified Chinese	584	NC	16.47	0	Alpha	0.85	NR	NR
58	Ceccarini M., 2015	[67]	YFAS‐16	Italian	88	C	NR	28.4	Alpha	0.9	NR	NR
59	Sevinçer G.M., 2015	[94]	YFAS	Turkish	171	C	36.13	27.4	Alpha	0.859	NR	NR
60	Innamorati M., 2015	[43]	YFAS	Italian	300	C	43.55	23	Alpha	0.83	NR	NR
61	Brunault P., 2014	[46]	YFAS	French	553	NC	28.9	NR	Kuder–Richardson 20	0.84	NR	NR
62	Gearhardt A.N., 2013	[54]	YFAS‐C 2.0	English	75	NC	8.32	57.3	Kuder–Richardson 20	0.78	NR	NR
63	Meule A., 2012 (Study 1)	[48]	YFAS	German	96	C	39.92	34.4	Alpha	0.82	NR	NR
64	Meule A., 2012 (Study 2)	[48]	YFAS	German	752	NC	23.13	22.6	Alpha	0.81	NR	NR
65	Gearhardt A.N., 2009	[28]	YFAS	English	353	NC	20.11	NR	Kuder–Richardson 20	0.86	NR	NR

*Notes*: NR = Not reported. Sample type: NC = Non‐Clinical Sample; C = Clinical Sample; M = Mixed Sample. KR‐20 and alpha coefficients are same coefficients; however, it calls KR‐20 when it is used for dichotomously scored items and it calls alpha when it is used for polytomously scored items. *ICC* = intraclass correlation coefficient.

The sample sizes across the 65 studies varied across studies. The median sample size was 451 participants. Some studies had extremely large sample sizes, such as the study by Niroumand Sarvandani et al (2024) with 9606 participants^58^ and the study by Nunes‐Neto et al (2018) with 7639 participants^59^ On the other hand, the smallest sample size was only 25 participants in the study by Druk et al (2020).^60^ Eight studies did not report the ages of the participants.^50^, ^61^, ^62^, ^63^, ^64^, ^65^, ^66^, ^67^ Of the remaining studies that reported ages, the median mean age across studies was 27.2 years. Most studies had adult samples, while some focused specifically on adolescents, such as the study by Horsager et al (2023) with a mean age of 15.15 years.^68^ The study with the youngest mean age was by Gearhardt et al (2013), at 8.32 years, while the oldest was the clinical sample from Manzoni et al (2021), with a mean age of 55.54 years.^54^ The percentage of males in the study samples was not reported for four of the studies.^28^, ^46^, ^61^, ^65^ In the remaining studies reporting sex distributions, the percentage of males ranged from 0% **i.e.** all‐female samples.^47^, ^69^, ^70^ to 57.3%.^54^ The median percentage of males across studies was 34.4%, indicating a female predominance in a typical study sample. The details are presented in Table 1.

Table 1 provides additional descriptive information on 65 studies that used various versions of the YFAS or their variants. The studies span different languages, including Spanish,^61^, ^62^, ^71^, ^72^ Persian,^58^, ^64^, ^70^, ^73^, ^74^, ^75^ Danish,^68^, ^76^, ^77^ Finnish,^78^ Portuguese,^45^, ^59^, ^65^, ^69^ Chinese,^47^, ^73^, ^79^, ^80^, ^81^ Arabic,^82^, ^83^ Czech,^63^, ^84^ Italian,^42^, ^43^, ^67^, ^85^, ^86^, ^87^ French,^46^, ^88^, ^89^ Russian,^60^ Malay,^90^, ^91^ Korean,^53^ Japanese,^92^ Turkish,^93^, ^94^ Hungarian,^95^ German,^48^, ^96^ and English.^28^, ^50^, ^51^, ^54^, ^66^, ^97^, ^98^, ^99^, ^100^

**FIGURE 2:**
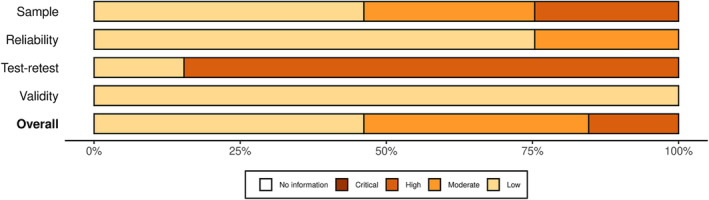
Summary plot of the assessment of the risk of bias.

**FIGURE 3:**
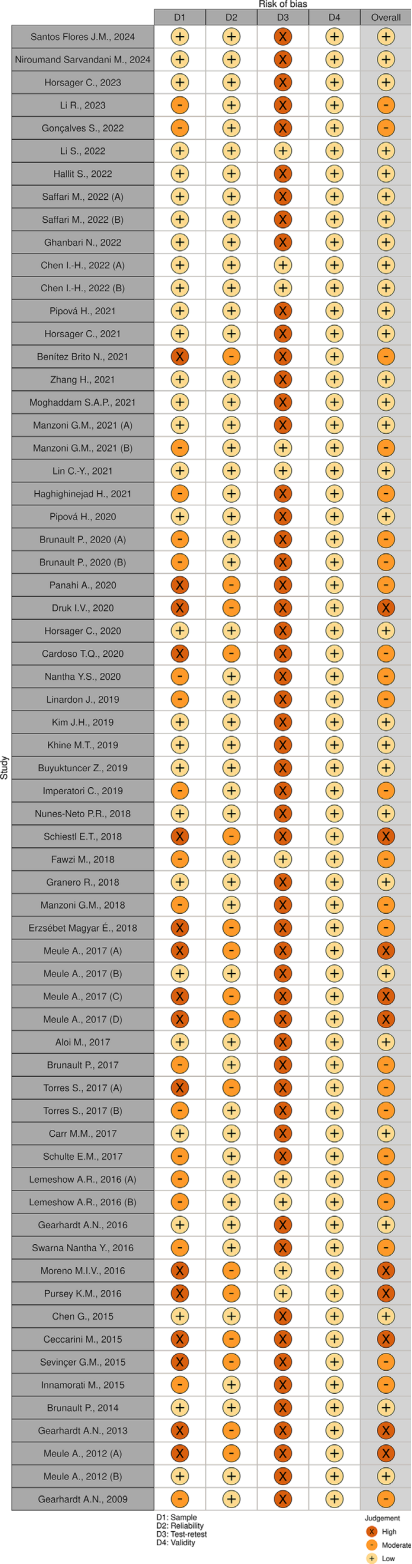
Traffic light plot of the assessment of the risk of bias.

The included studies exhibited varying levels of quality and risk of bias. While some studies demonstrated higher quality and lower risk of bias, others had limitations in terms of sample size, reliability, and validity (Figures 2 and 3). Among the included studies, the reliability of the measurements was assessed and categorized into three levels: low, moderate, and high. Most of the studies (63.6%) fell into the low‐reliability category, indicating potential limitations in the consistency and stability of the measurements used. A smaller proportion of the studies (22.7%) were classified as having moderate reliability, suggesting a relatively better level of consistency. A minority of the studies (12.1%) demonstrated high reliability, indicating strong consistency and stability in the measurements employed. Similarly, the test–retest reliability of the measurements was evaluated and classified into the same three categories: low, moderate, and high. Most studies (68.2%) were assigned to the low test–retest reliability category, implying potential fluctuations and inconsistencies in the measurements over time. A smaller percentage of the studies (16.7%) were categorized as having moderate test–retest reliability, indicating a relatively better level of stability over time. A minority of the studies (13.6%) demonstrated high test–retest reliability, suggesting strong stability and consistency in the measurements across repeated assessments.

Most studies reported the internal consistency of the scales using Cronbach's alpha or Kuder–Richardson 20 (KR‐20) coefficients. The alpha values ranged from 0.67 for the modified YFAS 2.0 in the study by Lemeshow et al (2016)^50^ to 0.95 for the YFAS 2.0 in the study by Gonçalves et al (2022).^69^ The KR‐20 values ranged from 0.69 for the YFAS‐C in the study by Kim et al (2019)^53^ to 0.93 for the YFAS 2.0 in the study by Ghanbari et al (2022).^73^

Some studies also reported test–retest reliability, with intraclass correlation coefficients (*ICC*s) ranging from 0.56 in the study by Moreno et al (2016)^72^ to 0.95 in the study by Fawzi et al (2018).^83^ Additionally, a few studies reported Cohen's kappa coefficients for test–retest reliability, with values of 0.73 and 0.79 reported by Lemeshow et al (2016).^50^

**FIGURE 4:**
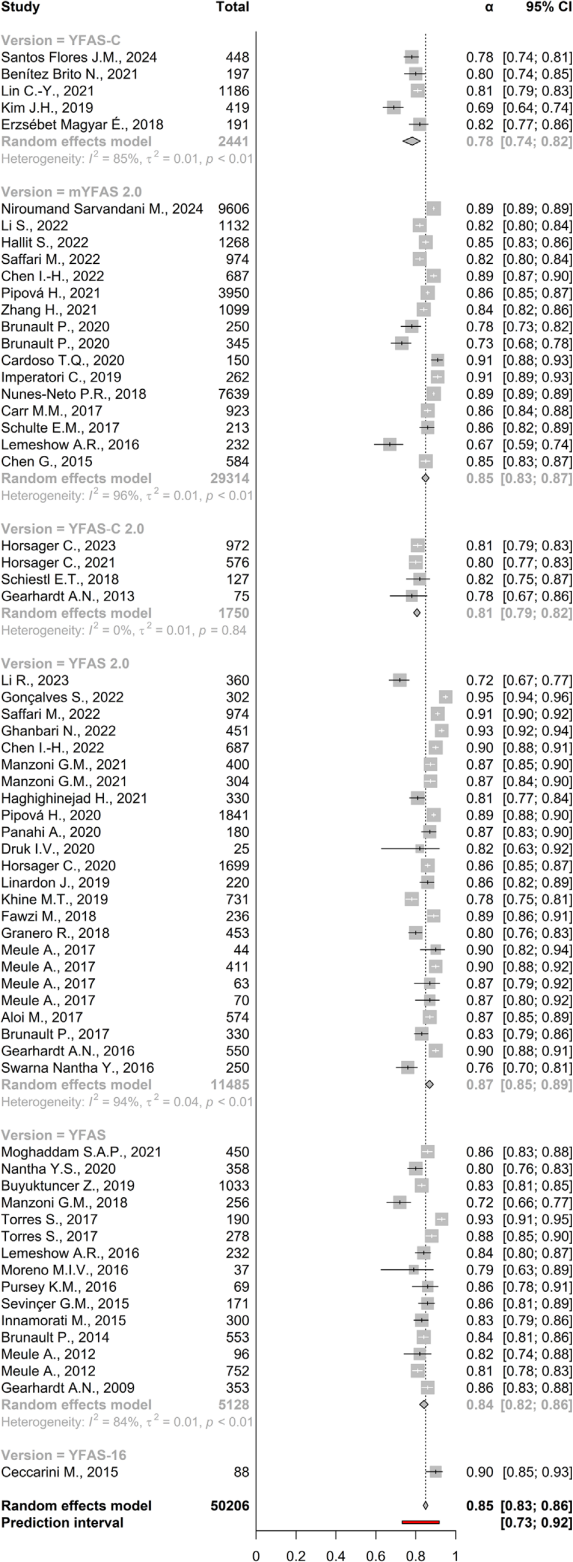
Meta‐analysis of the internal consistency of the Yale Food Addiction Scale (YFAS) and its variants.

**FIGURE 5:**
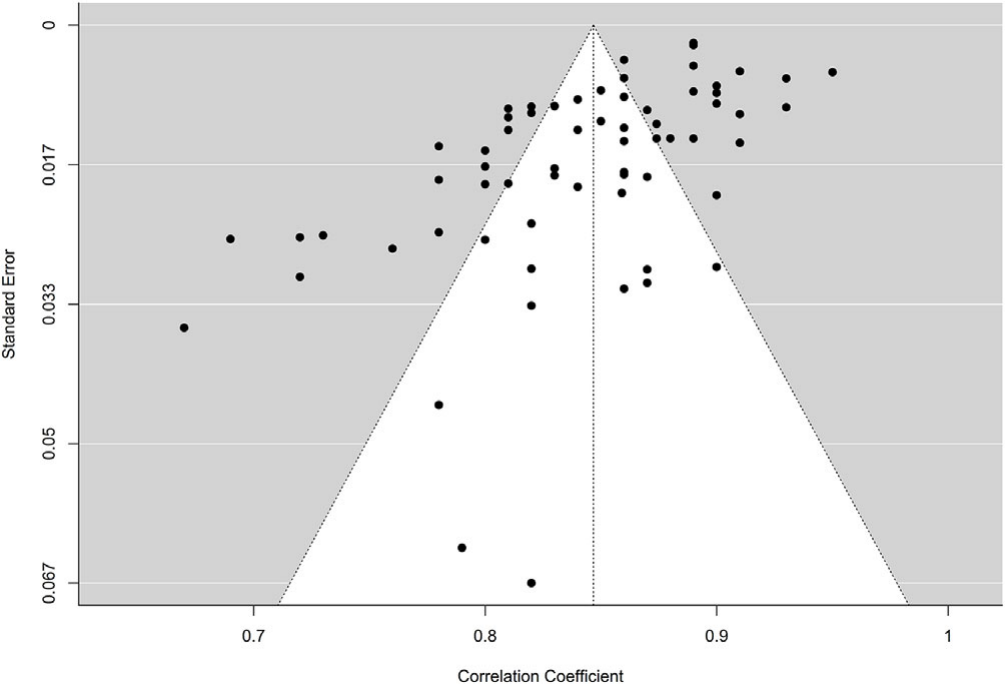
Funnel plot of the internal consistency of the Yale Food Addiction Scale (YFAS) and its variants.

A random‐effects meta‐analysis was conducted to estimate the average internal consistency reliability of the YFAS across 65 studies (total *n* = 50,206). The estimated pooled reliability coefficient from the random‐effects model as measured by Cronbach's alpha was 0.85 (95% CI: 0.83 to 0.86, *p* < 0.001), indicating high internal consistency on average. Heterogeneity tests revealed significant between‐study variability in reliability estimates (Q [df = 64] = 1107.98, *p* < 0.001, *I* ^*2*^ = 94.22%). The prediction interval, which accounts for this heterogeneity, ranged from 0.77 to 0.92, suggesting that despite variability, the YFAS demonstrated acceptable to excellent internal consistency across studies (Figure 4). Publication bias was assessed through several methods. Egger's regression test was significant (*p* < 0.001), indicating potential funnel plot asymmetry (Figure 5). The trim‐and‐fill method imputed 13 missing studies, suggesting the presence of publication bias. However, the fail‐safe N was extremely large at 9,243,607, meaning that it would require over 9 million missed null studies to nullify the overall high reliability. Tests revealed that the observed number of significant reliability estimates (65 out of 65) was exactly as expected given the average coefficient. Power analysis confirmed very high power (100%) to detect meta‐analytic reliability across studies. P‐uniform publication bias testing also indicated potential bias, but even when adjusting for this bias, the p‐uniform reliability estimates of *r* = 1.25 (95% CI: 1.21 to 1.29) remained excellent. Table 2 provides a summary of the meta‐analysis of the internal consistency of the YFAS and its variants.

**TABLE 2:** Meta‐analysis of the internal consistency of the Yale food addiction scale (YFAS) and its variants.

Statistic	Value
Random‐Effects Model Estimate	0.85
95% CI (Lower ‐ Upper Bound)	0.83‐0.86
Standard Error (se)	0.01
*Z* value	124.33
*p* value	< 0.001
Fisher r‐to‐z transformed Estimate	1.25
Fisher r‐to‐z transformed 95% CI (Lower ‐ Upper Bound)	1.21‐1.30
Heterogeneity Statistics	
*τ*	0.04
*τ* ^*2*^	00.0013 (SE = 6e‐04)
*I* ^*2*^	94.22%
*H* ^*2*^	17.31
*R* ^*2*^	
Degrees of Freedom (df)	64.00
Q	1107.98
p value	< 0.001
Number of Studies (k); Number of Sample (n)	*k* = 65; *n* = 50,206
Range of Observed Correlation Coefficients	0.6700‐0.9500
Percentage of Positive Estimates	100%
Publication Bias Assessment	
Fail‐Safe N	9243607.00 (*p* < 0.001)
Begg and Mazumdar Rank Correlation	‐0.20 (p = 0.021)
Egger's Regression	‐5.16 (*p* < 0.001)
Trim and Fill Number of Studies	13.00
Test of Excess Significance \ Significant Findings	
Observed Number of Significant Findings	65
Expected Number of Significant Findings	65
Observed Number/Expected Number	1.00
Test of Excess Significance \ Estimated Power of Tests (based on theta =	0.8468
Minimum	1.00
Q1	1.00
Median	1.00
Q3	1.00
Maximum	1.00
Test of Excess Significance	p = 1 (X2 = NA, df = 1)
Limit Estimate (where p = 0.1)	0.0866
Publication Bias Test p‐uniform	
Test Statistic	3.67
*p* value	< 0.001
Effect Size Estimation p‐uniform	
Effect Size Estimate	1.25
95% CI (Lower ‐ Upper Bound)	1.21‐1.29
*Z* value	‐13.96
Number of Significant Studies	65

Subgroup meta‐analysis by version showed that for the YFAS‐C version, five studies were included, with a random effects model estimating the reliability based on a combined sample size of 2441 participants. The analysis revealed a high level of heterogeneity (*I* ^*2*^ = 85%), indicating substantial variability across studies. The estimated reliability coefficient (*α*) ranged from 0.69 to 0.82, with a pooled estimate of 0.78 (95% CI: 0.74‐0.81). For the mYFAS 2.0 version, 15 studies were included, with a random effects model estimating the reliability based on a combined sample size of 29,314 participants. Similar to the YFAS‐C version, a high level of heterogeneity (*I* ^*2*^ = 96%) was observed. The estimated reliability coefficient ranged from 0.67 to 0.91, with a pooled estimate of 0.82 (95% CI: 0.80‐0.84). For the YFAS‐C 2.0 version, four studies were included, and no heterogeneity was detected (*I* ^*2*^ = 0%). The estimated reliability coefficient ranged from 0.72 to 0.82, with a pooled estimate of 0.80 (95% CI: 0.77‐0.83). For the YFAS 2.0 version, 24 studies were included, with a random effects model estimating the reliability based on a combined sample size of 11,485 participants. A high level of heterogeneity (*I* ^*2*^ = 94%) was observed. The estimated reliability coefficient ranged from 0.59 to 0.89, with a pooled estimate of 0.86 (95% CI: 0.84‐0.88). Finally, for the YFAS and YFAS‐16 versions, 15 and 16 studies, respectively, were included. The estimated reliability coefficients for these versions ranged from 0.67 to 0.89 and from 0.72 to 0.93, respectively (Figure 4).

Meta‐regression (age and sex) and subgroup analyses (population type and language) were used to explore potential reliability moderators. Meta‐regression models showed that neither age nor sex appeared to moderate the overall internal consistency of the YFAS or its variants. Subgroup analysis by population revealed no difference between non‐clinical vs clinical vs mixed sample types. For non‐clinical samples (*k* = 46, *n* = 43,492), the observed correlation coefficients ranged from 0.67 to 0.95. The estimated average correlation coefficient based on the random‐effects model was 0.84 (95% CI: 0.81 to 0.86). A 95% prediction interval for the true outcomes is given by 0.78 to 0.92. For clinical samples (*k* = 13, *n* = 3941), the observed correlation coefficients ranged from 0.73 to 0.93. The estimated average correlation coefficient based on the random‐effects model was 0.84 (95% CI: 0.81 to 0.87). A 95% prediction interval for the true outcomes is given by 0.74 to 0.95. For mixed samples (*k* = 6, *n* = 2773), the observed correlation coefficients ranged from 0.80 to 0.87. The estimated average correlation coefficient based on the random‐effects model was 0.84 (95% CI: 0.81 to 0.87). A 95% prediction interval for the true outcomes is given by 0.78 to 0.90. Subgroup analysis by language also revealed no differences between languages. Specifically, languages with five or more studies included Chinese, English, German, Italian, Persian, and Portuguese, and the respective internal consistencies were 0.85 (95% CI: 0.81 to 0.89), 0.84 (95% CI: 0.82 to 0.87), 0.86 (95% CI: 0.82 to 0.90), 0.86 (95% CI: 0.83 to 0.89), 0.86 (95% CI: 0.83 to 0.90), and 0.90 (95% CI: 0.88 to 0.94).

**FIGURE 6:**
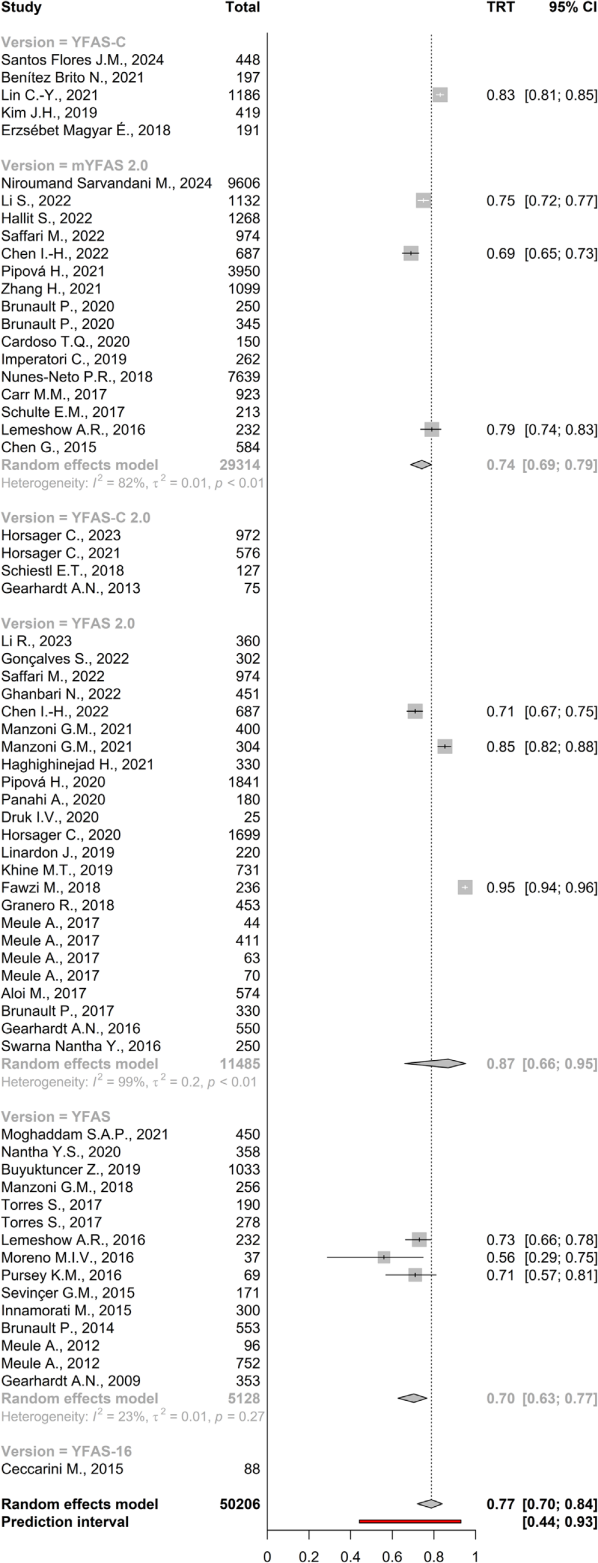
Meta‐analysis of the test–retest of the Yale Food Addiction Scale (YFAS) and its variants.

**FIGURE 7:**
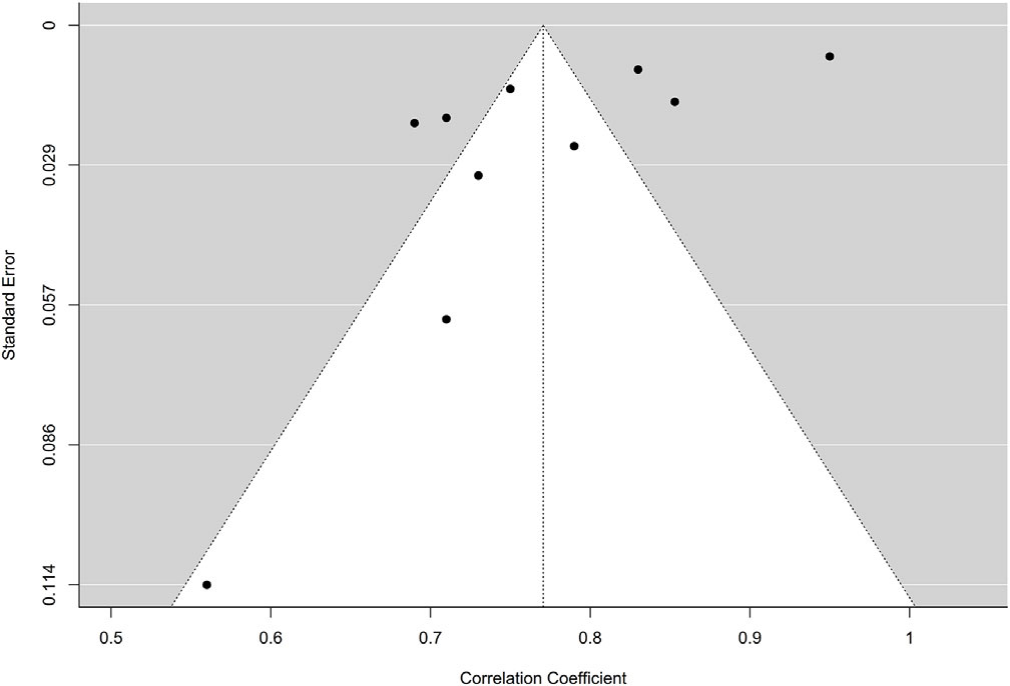
Funnel plot of the test–retest of the Yale Food Addiction Scale (YFAS) and its variants.

A random‐effects meta‐analysis was conducted on 10 studies (total *n* = 4802) to estimate the average test–retest reliability of the YFAS. The estimated pooled test–retest correlation coefficient was 0.77 (95% CI: 0.70 to 0.84, *p* < 0.001), indicating large temporal stability on average (Figure 6). Publication bias was assessed using the funnel plot method (Figure 7). Significant heterogeneity was present across studies in the test–retest estimates (Q [df = 9] = 456.95, *p* < 0.001, *I* ^*2*^ = 98.03%). The prediction interval, which accounts for this high degree of heterogeneity, ranged from 0.52 to 1.02. This suggests that while the average test–retest correlation was large, there was considerable variability across studies, with some finding much lower or even negative test–retest reliability coefficients. One study^83^ was identified as a potential outlier. No studies based on standard diagnostics were overly influential. While some indicators suggested potential publication bias, the fail‐safe N was extremely large at 91,568, meaning that more than 91,000 missed null studies would be required to nullify the overall large test–retest effect. The number of significant test–retest findings (10 out of 10) was exactly as expected. The power to detect the meta‐analytic test–retest coefficient across studies was 100%. After adjusting for potential publication bias, the test–retest estimate remained large at 0.97 (95% CI: 0.89 to 1.12). Table 3 provides a summary of the meta‐analysis of the test–retest reliability of the YFAS and its variants.

**TABLE 3:** Meta‐analysis of the test–retest reliability of the Yale food addiction scale (YFAS) and its variants.

Statistic	Value
Random‐Effects Model Estimate	0.77
95% CI (Lower ‐ Upper Bound)	0.70‐0.84
Standard Error (se)	0.03
*Z* value	24.87
*p* value	< 0.001
Fisher r‐to‐z transformed Estimate	1.07
Fisher r‐to‐z transformed 95% CI (Lower ‐ Upper Bound)	0.91‐1.22
Heterogeneity Statistics	
*τ*	0.11
*τ* ^*2*^	0.0111 (SE = 0.0082)
*I* ^*2*^	98.03%
*H* ^*2*^	50.77
*R* ^*2*^	
Degrees of Freedom (df)	9.00
Q	456.95
p value	< 0.001
Number of Studies (k); Number of Sample (n)	*k* = 10; *n* = 4802
Range of Observed Correlation Coefficients	0.5600‐0.9500
Percentage of Positive Estimates	100%
Publication Bias Assessment	
Fail‐Safe N	91568.00 (*p* < 0.001)
Begg and Mazumdar Rank Correlation	0.02 (p = 1.000)
Egger's Regression	‐2.58 (p = 0.033)
Trim and Fill Number of Studies	3.00
Test of Excess Significance \ Significant Findings	
Observed Number of Significant Findings	10
Expected Number of Significant Findings	10
Observed Number/Expected Number	1.00
Test of Excess Significance \ Estimated Power of Tests	based on theta = 0.7706
Minimum	1.00
Q1	1.00
Median	1.00
Q3	1.00
Maximum	1.00
Test of Excess Significance	p = 1 (X2 = NA, df = 1)
Limit Estimate (where p = 0.1)	NA
Publication Bias Test p‐uniform	
Test Statistic	1.40
*p* value	0.080
Effect Size Estimation p‐uniform	
Effect Size Estimate	0.97
95% CI (Lower ‐ Upper Bound)	0.89‐1.12
*Z* value	‐5.47
Number of Significant Studies	10

## 4 DISCUSSION

The current meta‐analysis examined the reliability of the YFAS and its variants across demographic groups and regions. The findings reveal the consistency and robustness of the YFAS in detecting FA across groups. The YFAS thus appears to be reliable. We have concluded that the internal consistency of the YFAS is good, with an estimated pooled reliability coefficient *α* of 0.85 across 65 studies. Despite high‐reliability estimates of variations between investigations, the prediction interval implies good internal consistency. This reliability is crucial for its use in researching compulsive eating behaviors and obesity. The high internal consistency and test–retest reliability suggest that the YFAS can consistently identify symptoms of food addiction, which may indicate maladaptive eating patterns that contribute to obesity. The reliability of the YFAS has important implications for understanding the complex causes of eating disorders and obesity. By providing a consistent measure of FA symptoms, the YFAS can help researchers and clinicians better identify individuals who may be prone to compulsive overeating. This, in turn, can inform the development of targeted interventions and treatments for those struggling with obesity and related eating disorders. The strong psychometric properties of the YFAS support its use in longitudinal studies that examine the progression of compulsive eating behaviors and their relationship to obesity over time. Such research is vital for developing effective prevention strategies and early interventions.

Reliability estimates may vary due to sample characteristics, cultural variables, and administration methods. For instance, study sample sizes, age ranges, and gender compositions may affect YFAS dependability. Language translations and cultural adjustments of the scale may also affect dependability estimates. The current meta‐analysis included only 10 of 65 studies that reported test–retest reliability. Based on these studies, we found that the YFAS's test–retest reliability was highly stable, with an estimated pooled correlation coefficient of 0.77. However, test–retest estimates showed significant heterogeneity among studies, indicating variability in response stability. The YFAS test–retest reliability was high despite this fluctuation. This meta‐analysis has important implications for research and therapeutic practice. The good internal consistency and test–retest reliability of the YFAS suggest that it can detect FA in various groups. Researchers and clinicians can confidently utilize the YFAS to identify FA and devise tailored interventions.

FA is closely linked to obesity, with studies highlighting the prevalence and clinical significance of this relationship. Research has identified distinct profiles of FA symptoms among individuals with obesity, with some showing heightened severity of psychopathology.^33^ Emotional eating, a common trigger for self‐regulation failure, is associated with obesity and FA, indicating a connection between affect regulation and addictive eating behaviors.^101^ Furthermore, rates of co‐occurrence between FA and problematic substance use, such as alcohol, smoking, and cannabis, suggest a potential addictive‐like eating phenotype, emphasizing the addictive nature of FA and its association with obesity.^102^ Therapeutic approaches targeting emotional coping mechanisms, cognitive restriction, and time‐restricted feeding have shown promise in managing FA and obesity, underscoring the importance of tailored interventions for individuals with these conditions.^103^

FA can be explicitly measured using a few standardized scales and questionnaires^104^ The YFAS and the Addiction‐like Eating Behavior Scale (AEBS) assess eating behaviors resembling addiction^105^ The YFAS, based on criteria for substance use disorders from the DSM, evaluates symptoms such as impaired control over eating and continued consumption despite negative consequences.^106^ In contrast, the AEBS focuses on observable behaviors linked to addiction‐like eating without relying on criteria for substance use disorders.^106^ This behavior‐focused assessment may address limitations associated with directly applying substance addiction criteria to eating behaviors.^106^ Other scales, while not specifically designed for FA, provide valuable insights into related concepts.^104^ These include the Eating Behaviors Questionnaire (EBQ),^107^ which measures addictive eating behaviors; the Food Cravings Questionnaire (FCQ),^108^ which assesses various aspects of food cravings; and the Power of Food Scale (PFS),^109^ which evaluates the psychological impact of living in food‐abundant environments. Thus, researchers and clinicians can use these scales to evaluate related behaviors and complement more targeted FA measures.

The integration of FA assessment tools like the YFAS into multidisciplinary obesity care presents both opportunities and challenges.^110^ While the YFAS offers a structured approach to identifying addictive‐like eating behaviors, its clinical utility is tempered by ongoing debates about the construct validity of FA itself.^111^ For psychologists and psychiatrists, the YFAS can provide valuable insights into patients' eating patterns and associated distress, potentially guiding the application of cognitive‐behavioral therapies.^112^ However, the frequent co‐occurrence between FA and other eating disorders or psychiatric conditions may complicate diagnosis and treatment planning.^113^ Nonetheless, the identification of FA has direct clinical implications for conditions with frequent co‐occurrence. For example, among individuals with binge eating disorder, the presence of FA has been linked to poorer treatment outcomes.^114^ Bariatric surgeons may find the high prevalence of FA in surgical candidates noteworthy, but current evidence does not clearly link pre‐surgical FA status to post‐operative outcomes, perhaps limiting its use in surgical decision‐making.^106^, ^115^ Nutritionists may leverage YFAS data to tailor dietary interventions, particularly in addressing the consumption of highly processed, palatable foods often associated with FA.^116^ The addictive‐like properties of these foods may necessitate more intensive or supportive approaches to dietary change.^117^

Based on our review the reliability of FA assessment and its clinical implications vary across disciplines.^117^ While the YFAS offers a standardized measure, its interpretation and application in clinical practice require careful consideration.^118^ The lack of DSM‐5 recognition for FA as a distinct diagnosis may lead to inconsistencies in how different team members conceptualize and address FA‐related behaviors.^119^ Nevertheless, the high prevalence of FA symptoms in obesity populations suggests it may be a relevant factor for many individuals, underscoring the need for a holistic, multidisciplinary approach.^120^ The association of FA with various psychosocial factors emphasizes the importance of comprehensive assessment and treatment planning.^120^ As research in this field evolves, all multidisciplinary team members should stay informed about emerging evidence to guide their practice.^121^ The potential role of FA in weight re‐gain and poorer weight loss outcomes highlights the importance of long‐term follow‐up and support from all members of multidisciplinary teams.

In the context of research, the debate over appropriate reliability thresholds is particularly relevant.^122^ While a Cronbach's alpha of 0.70 is generally considered acceptable for research purposes, especially with large datasets, this threshold may be insufficient for individual clinical assessment.^122^, ^123^ For research aimed at understanding population‐level trends or group differences in FA, an alpha of 0.70 can provide meaningful insights without compromising the overall validity of the findings.^123^ However, when considering the use of FA scales for individual patient assessment or diagnosis, a higher reliability threshold (e.g., 0.80 or 0.90) would be more appropriate to ensure accurate classification and minimize misdiagnosis risks.^106^ This distinction is noteworthy in FA studies, particularly as scales like the YFAS are used not only in research settings but also increasingly in clinical contexts.^111^ Our meta‐analysis primarily focused on research applications, where the 0.70 threshold is often deemed sufficient.^122^

To illustrate, using a hypothetical 10% prevalence of FA, a scale with *α* = 0.70 might correctly identify 70% of true cases with 96.7% specificity, whereas a scale with *α* = 0.90 could improve this to 90% sensitivity and 98.9% specificity. As FA research evolves, researchers should develop more reliable measures for clinical use, clearly differentiate between research and clinical reliability standards, and conduct studies specifically examining the impact of reliability on diagnostic accuracy.

Nevertheless, we acknowledge that for potential clinical applications of FA measures, particularly when making decisions about individual patients, researchers and clinicians should aim for higher reliability standards.^37^, ^104^ Notably, our overall reliability estimate is about 0.85, suggesting it may be excellent for both research and clinical settings. Future studies might benefit from explicitly differentiating between reliability requirements for research versus clinical purposes, especially as the field of FA continues to bridge research findings with clinical practice.

Suggested best practices for administering and interpreting the YFAS in the context of FA and obesity management may optimally involve a comprehensive and nuanced approach.^37^, ^104^ Clear instructions are important, and clinicians should be available to clarify potential questions, as misinterpretation of items may impact results. When interpreting YFAS scores, clinicians should consider them within the broader context of the patient's medical history, psychological profile, and current life circumstances.^44^ Clinicians should account for potential confounding factors such as recent dietary changes, medication use, or co‐occurring psychiatric conditions.^124^ It is advisable to use the YFAS alongside other validated measures of eating behavior and psychological functioning to create a more complete clinical picture.^125^ Clinicians should avoid over‐interpreting YFAS results, recognizing that while it can indicate problematic eating patterns, it does not currently constitute a formal psychiatric diagnosis.^126^ In obesity management, YFAS results can guide treatment planning by identifying specific problematic behaviors or triggers that targeted interventions can address.^126^ However, clinicians should communicate results to patients sensitively, using non‐stigmatizing language and framing FA as a manageable condition rather than a personal failing.^127^ Regular reassessment using the YFAS helps track changes over time and evaluate treatment effectiveness, but clinicians should remain mindful of potential practice effects with repeated administrations.^126^

The YFAS may has several limitations when used with bariatric patients.^115^ Pre‐surgical dietary restrictions and post‐surgical physiological changes may skew responses, preventing an accurate assessment of altered experiences of hunger and cravings.^115^ Furthermore, behaviors that may be deemed “addictive” could actually reflect normal or necessary behaviors after surgery, leading to misclassification.^128^ Psychological adjustments related to body image and food relationships also influence responses.^128^ The scale's limited long‐term validation in this population and the lack of surgery‐specific norms may lead to inaccurate comparisons and the over‐pathologizing of typical post‐surgical eating patterns.^129^ Thus, clinicians should exercise caution and use the YFAS alongside tailored tools and comprehensive clinical evaluations with certain populations such as bariatric patients.

The current analysis offers a robust synthesis of the psychometric properties of the YFAS across studies. The results generally support the reliability of the YFAS, particularly with dimensional scoring approaches. However, the subscale reliabilities and impacts of moderators warrant continued examination, especially as the scale evolves through future revisions and applications expand to novel populations.

Limitations include the reliance on published studies only, although publication bias analyses did not reveal substantial impacts. The analysis also focused specifically on internal consistency reliability coefficients, as reported; future investigations could explore other psychometric factors, such as validity evidence and structural analyses of scale dimensionality.

In conclusion, this REGEMA provided an overarching quantitative synthesis of the reliability characteristics of YFAS scores. The results highlighted the measure's overall adequate reliability properties while also delineating key moderating factors that may alter score fidelity across applications. These findings offer guidance for scale usage while underscoring the need for continued scrutiny as the addictive eating construct and YFAS undergo further conceptual and psychometric maturation.

## AUTHOR CONTRIBUTIONS

Conceptualization, Haitham Jahrami, Waqar Husain, and Amir H. Pakpour; methodology, Haitham Jahrami, Waqar Husain, and Amir H. Pakpour; software, Haitham Jahrami; validation, Haitham Jahrami, Waqar Husain, and Amir H. Pakpour; formal analysis, Haitham Jahrami; investigation, Haitham Jahrami, Waqar Husain, and Amir H. Pakpour; resources, Haitham Jahrami and Waqar Husain; data curation, Haitham Jahrami, and Waqar Husain; writing—original draft preparation, Haitham Jahrami, Waqar Husain, Khaled Trabelsi, Achraf Ammar, Seithikurippu R. Pandi‐Peruma, Zahra Saif, Marc N. Potenza, Chung‐Ying Lin, and Amir H. Pakpour; writing—review and editing, Haitham Jahrami, Waqar Husain, Khaled Trabelsi, Achraf Ammar, Seithikurippu R. Pandi‐Peruma, Zahra Saif, Marc N. Potenza, Chung‐Ying Lin, and Amir H. Pakpour; visualization, Haitham Jahrami; supervision, Haitham Jahrami; project administration, Haitham Jahrami, Waqar Husain and Amir H. Pakpour. All authors have read and agreed to the published version of the manuscript.

## CONFLICT OF INTEREST

Dr. Potenza has consulted for Opiant Therapeutics, Baria‐Tek, and Boehringer Ingelheim; has been involved in a patent application with Yale University and Novartis; has received research support from Mohegan Sun Casino, Children and Screens, and the Connecticut Council on Problem Gambling; has participated in surveys, mailings or telephone consultations related to drug addiction, impulse‐control disorders or other health topics; has consulted for and/or advised gambling, non‐profit and legal entities on issues related to impulse control, internet use, and addictive disorders; has performed grant reviews for research‐funding agencies; has edited journals and journal sections; has given academic lectures in grand rounds, CME events and other clinical or scientific venues; and has generated books or book chapters for publishers of mental health texts. The other authors do not report disclosures.

## INSTITUTIONAL REVIEW BOARD STATEMENT

Not applicable.

## INFORMED CONSENT STATEMENT

Not applicable.

## Data Availability

The current study utilized secondary data, i.e., previously published articles (references provided herein, all data available in Table 1 and on The Open Science Framework [OSF] Identifier: DOI 10.17605/OSF.IO/G2BYU URL https://osf.io/g2byu/).
